# Gail Hamilton

**Published:** 1866-06

**Authors:** 


"Gail Hamilton has in press of her publishers, Messrs Ticknor & Fields, Boston, a new volume specially adapted to summer reading, and bearing the taking title of "Summer Rest." Most of the articles in this volume are now for the first time printed, and will be found equal to any of the author's most brilliant essays. Halicarnassus appears again on the carpet; and his exploits in the way of gardening and other domestic matters are made very amusing. Gail Hamilton is never dull. Possessed of a sharp and ready wit, speaking boldly, and that too upon topics wherein women have been supposed to have but little interest, she has already gathered about her an audience, which, by its hearty appreciation of her writings, attests the truth of many of her convictions. The success of her various volumes of essays has been without a parallel; in fact she is the most successful writer of the day."

